# Supplementary material for: The Emerging Evidence for a Protective Role of Fucoidan from Laminaria japonica in Chronic Kidney Disease-Triggered Cognitive Dysfunction
Source: Mar Drugs. 2022 Apr 7;20(4):258. doi: 10.3390/md20040258 (PMC9025131; doi:10.3390/md20040258)
Supplement: Supplementary file 1 [file marinedrugs-20-00258-s001.zip › marinedrugs-1656916-supplementary.pdf]

Supplementary materials

The Emerging Evidence for A Protective Role of Fucoidan from *Laminaria japonica* in Chronic Kidney Disease-Triggered Cognitive Dysfunction

Zhihui Ma [1,†], Zhiyou Yang [1,2,†,*], Xinyue Feng [1], Jiahang Deng [1], Chuantong He [1], Rui Li[1], Yuntao Zhao [1], Yuewei Ge [3], Yongping Zhang [1], Cai Song [1], Saiyi Zhong [1,2]

[1] Guangdong Provincial Key Laboratory of Aquatic Product Processing and Safety, Guangdong Province Engineering Laboratory for Marine Biological Products, Guangdong Provincial Engineering Technology Research Center of Seafood, Key Laboratory of Advanced Processing of Aquatic Product of Guangdong Higher Education Institution, College of Food Science and Technology, Guangdong Ocean University, Zhanjiang 524088, China; mazhihui@stu.gdou.edu.cn (Z.M.); 2112003030@stu.gdou.edu.cn (X.F.); 2112003070@stu.gdou.edu.cn (J.D.); 2112103046@ stu.gdou.edu.cn (C.H.); lirui@gdou.edu.cn (R.L.); zhaoyt@gdou.edu.cn (Y.Z.); zhangyp@gdou.edu.cn (Y.Z.); cai.song@dal.ca (C.S.); zhongsy@gdou.edu.cn (S.Z.)

[2] Collaborative Innovation Center of Seafood Deep Processing, Dalian Polytechnic University，Dalian 116034, China

[3] Key Laboratory of Digital Quality Evaluation of Chinese Materia Medica of State Administration of TCM, Guangdong Pharmaceutical University, Guangzhou 510006, China; geyuewei@gdpu.edu.cn (Y.G.)

*Correspondence: zyyang@gdou.edu.cn (Z.Y.); Tel.: +86-759-2396046

†These authors contributed equally to this work.

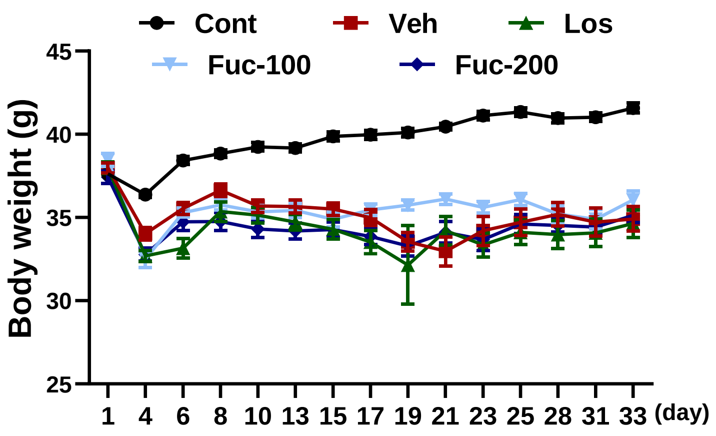

Figure S1. Effects of fucoidan on body weight in adenine-induced CKD mice. ICR mice (male, 8-week-old) were orally co-administered with fucoidan (Fuc), Losartan (Los) or vehicle (saline), and adenine (0.25% contained diets) for 33 days.

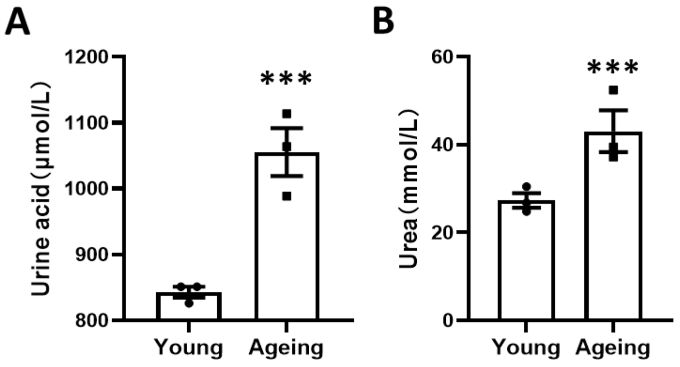

Figure S2. Effects of fucoidan on renal indexes in ageing renal failure mice. The urine was collected from young C57BL/6 mice (male, 3-month-old) and old C57BL/6 mice (male, 24-month-old), and were applied for ELISA analysis using urine acid (A) and urea (B) kits.

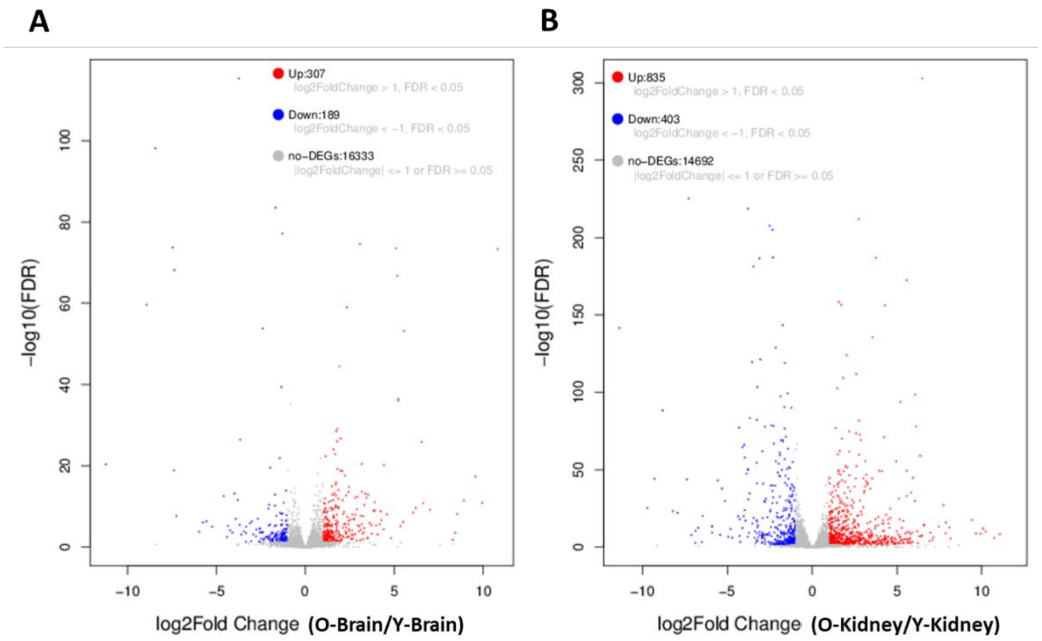

Figure S3. Volcano map of differential genes in the brain and kidney of CKD mice. The aged-CKD mice (male, 24-month-old) and young mice (male, 3-month-old) were sacrificed, and the brain and kidney were dissected after perfusion with saline. The tissues were then subjected to RNA-seq analysis. (A) Differential genes expressed in the brain between aged-CKD and young mice. (B) Differential genes expressed in the kidney between aged-CKD and young mice. The data are expressed as mean ± SEM (n = 3).

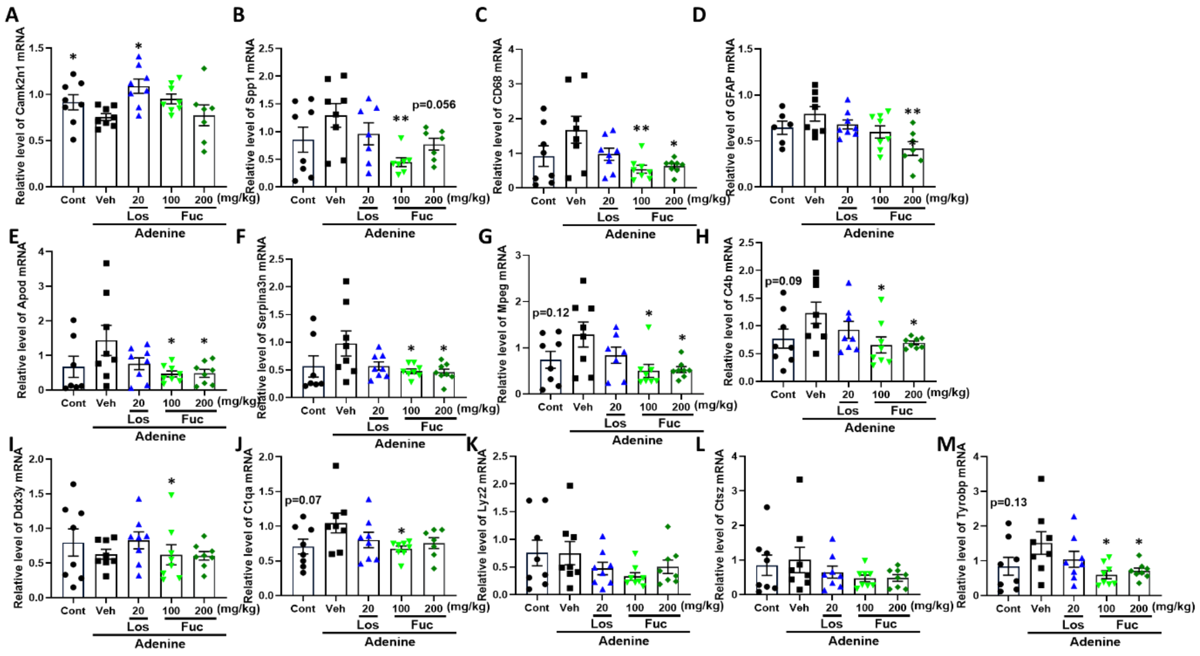

Figure S4. Effects of fucoidan (Fuc) on adenine-induced mRNA expression of differential genes in hippocampus. Camk2n1 (A), Spp1 (B), CD68 (C), GFAP (D), Apod (E), Serpina3n (F), Mpeg (G), C4b (H), Ddx3y (I), C1qa (J), Lyz2 (K), CTSZ (L), and Tyrobp (M) were determined by mRNA qPCR analysis. *p < 0.05, **p < 0.01 vs. vehicle group (Veh), one-way ANOVA post hoc Dunnett’s test.

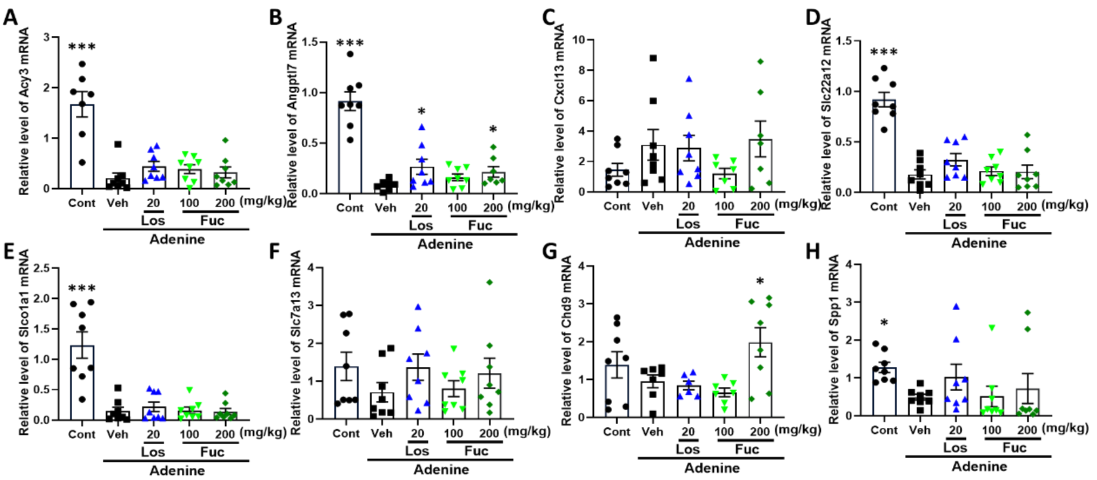

Figure S5. Effects of fucoidan (Fuc) on adenine-induced mRNA expression of differential genes in kidney. Acy3 (A), Angptl7 (B), Cxcl13 (C), Slc22a12 (D), Slco1a1 (E), Slc7a13 (F), Chd9 (G), and Spp1 (H) were determined by mRNA qPCR analysis. *p < 0.05, ***p < 0.001 vs. vehicle group (Veh), one-way ANOVA post hoc Dunnett's test.

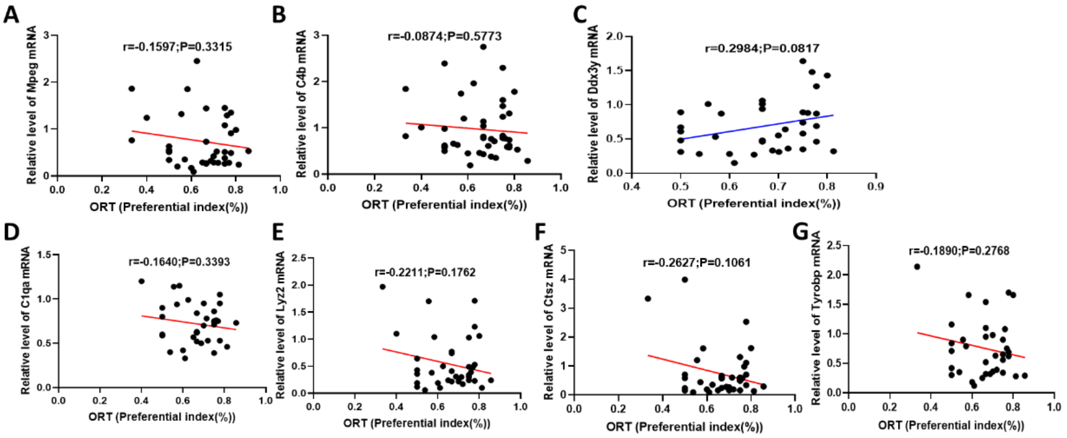

Figure S6. Pearson correlation analysis between hippocampus gene expression and cognitive behavior. The mRNA qPCR analysis of Mpeg (A), C4b (B), Ddx3y (C), C1qa (D), Lyz2 (E), Ctsz (F), and Tyrobp (G) were performed.

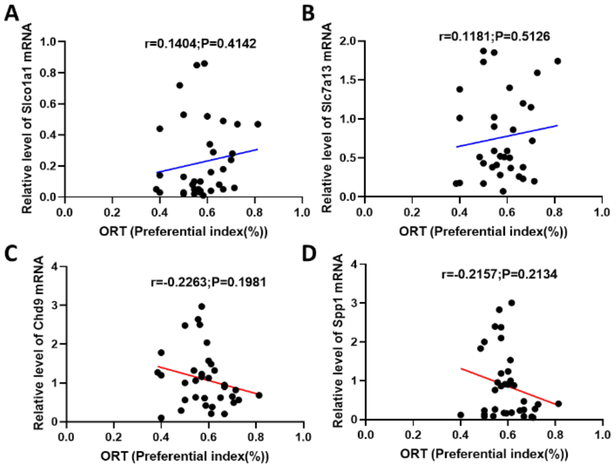

Figure S7. Pearson correlation analysis between kidney gene expression and cognitive behavior. The mRNA qPCR analysis of Slco1a1 (A), Slc7a13 (B), Chd9 (C), and Spp1 (D) were performed.

Table S1. Design of amplification primers for genes related to oxidative damage and inflammatory factors in epigenetic kidney and hippocampus.

Primer	Sequence (5' to 3') F	Sequence (5' to 3') R
GSK-3β	ATGGCAGCAAGGTAACCACAG	TCTCGGTTCTTAAATCGCTTGTC
Nrf2	TCCGCTGCCATCAGTCAGTC	ATTGTGCCTTCAGCGTGCTTC
HO-1	CCCAAAACTGGCCTGTAAAA	CGTGGTCAGTCAACATGGAT
NQO1	AGGATGGGAGGTACTCGAATC	AGGCGTCCTTCCTTATATGCTA
TNFα	GACGTGGAACTGGCAGAAGAG	TTGGTGGTTTGTGAGTGTGAG
IL1β	GCAACTGTTCCTGAACTCAACT	ATCTTTTGGGGTCCGTCAACT
IL4	GGTCTCAACCCCCAGCTAGT	GCCGATGATCTCTCTCAAGTGAT
iNOS	CTGCAGCACTTGCATCAGGAACCTG	GAGTAGCCTGTGTGCACCTGGAA
Arg1	TAACCTTGGCTTGCTTCGGAACTC	TGGCGCATTCACAGTCACTTAGG
CD206	TCTTTGCCTTTCCCAGTCTCC	TGACACCCAGCGGAATTTC
TGFβ	CTCCCGTGGCTTCTAGTGC	GCCTTAGTTTGGACAGGATCTG
Camk2n1	GGAGCAAGCGCGTTGTTATT	CAGCCCGCCACTCTTCTTAT
Spp1	CCTTGCTTGGGTTTGCAGTC	TGGTCGTAGTTAGTCCCTCAGA
Cd68	TCAGAGCCCGAGTACAGTCT	GCCATGAATGTCCACTGTGC
GFAP	GCTGCGTATAGACAGGAGGC	TCTCCTCCTCCAGCGATTCA
Apod	TATCAAAGGGCAAGGGCCTG	GCTCACTGTCAGTTTCTCTCAG
Serpina3n	ACCCAGGGGATGATCAAGGA	GGCACCTTCCATTTGGCTTT
Mpeg1	AGCCTTCTGACAGAGTCTTGTT	CCCGTTTCTCCAAGAGGCTT
C4b	GCCCCATGTCCTGTTGTACT	AGAGCACTTGTGATCAGGGC
Ddx3y	GGGTCTGTGATAAGGACAGTTCA	CACGACCACCAATACCATCATAG
C1qa	TGCTGACCATGACCCTAGTAT	AGCAGCTCCTGGCTCC
Lyz2	TGAACGTTGTGAGTTTGCCAG	AGCTAAACACACCCAGTCGG
Ctsz	GTGATTACGGCTCCCTGTCC	GCCATTATCCCGCAGCTGAT
Tyrobp	GTCGCCTTATCAGGAGCTTCA	GGATCCGGGCATCAGGC
Acy3	GCTCGTACCAGCAGCTTAGG	TCAAGACACCAGCAGCCAAA
Slc22a12	GAGGAACCAAGCAGGGACAA	CCAAAGGCAAACCAGCACAG
Cxcl13	CTCTCCAGGCCACGGTATTC	TGATGTTTAGACCGACAACAGT
Angptl7	GCCCTGAGCTAGAGGTGTTC	CCAGAAGTCACCTCGGATGC
Slco1a1	ACAACAAGCTGCAGTACTTTTTA	TCAGATTTGACACACCTCAGAAG
Slc7a13	GGACTGCCTCTGGTAACTGTC	AGAGCAACAGCATCTGAAGAG
Chd9	TGTTGTTAAACAGTCTGGGTTTC	GCCAGCAGGAACCGC
β-actin	CATCCGTAAAGACCTCTATGCCAAC	ATGGAGCCACCGATCCACA
